# A majestic morning walk…!

**Published:** 2008

**Authors:** Sanjeev V. Thomas

**Affiliations:** Professor of Neurology and Editor, Annals of Indian Academy of Neurology, Department of Neurology, SCTIMST, Trivandrum, India. E-mail: sanjeev@sctimst.ac.in



